# Intestinal absorption of BCS class II drugs administered as nanoparticles: A review based on *in vivo* data from intestinal perfusion models

**DOI:** 10.5599/admet.881

**Published:** 2020-09-17

**Authors:** David Dahlgren, Erik Sjögren, Hans Lennernäs

**Affiliations:** Department of Pharmaceutical Biosciences, Translational Drug Discovery and Development, Uppsala University, Sweden

**Keywords:** Nano particle, nano drug delivery, nanomedicines, intestinal perfusion, pharmaceutical development

## Abstract

An established pharmaceutical strategy to increase oral drug absorption of low solubility–high permeability drugs is to create nanoparticles of them. Reducing the size of the solid-state particles increases their dissolution and transport rate across the mucus barrier and the aqueous boundary layer. Suspensions of nanoparticles also sometimes behave differently than those of larger particles in the fed state. This review compares the absorption mechanisms of nano- and larger particles in the lumen at different prandial states, with an emphasis on data derived from in vivo models. Four BSC class II drugs—aprepitant, cyclosporine, danazol and fenofibrate—are discussed in detail based on information from preclinical intestinal perfusion models.

## Introduction

Rate and extent of drug absorption and bioavailability are critical pharmacokinetic (PK) parameters for oral pharmaceutical products. These parameters are determined and characterized in drug discovery and in preclinical and clinical development of a drug product. Successful design and development of any oral drug product requires understanding the complex interplay between the pharmacokinetic parameters, manufacturing methods, pharmaceutical excipients, and biopharmaceutics, as well as potency (the major driver for drug discovery), pharmacology, safety, and toxicity. Gastrointestinal (GI) absorption and bioavailability are both affected by a number of pharmaceutical, biopharmaceutical and physiological processes. Despite comprehensive knowledge about drug-product related and physiological properties in GI absorption, many drug candidates and drugs have suboptimal biopharmaceutical properties for oral dosing [1, 2]. Several challenging drug candidates are listed in the biopharmaceutical classification system (BCS) as class II, III, or IV, for which absorption is limited by solubility properties in GI luminal fluids and/or intestinal permeability [3]. Limitations in any one of the main BCS parameters may reduce the rate and fraction dose absorbed (*f*_abs_) from conventional, oral dosage forms intended for immediate release in the GI lumen. Drugs with low solubility and/or low intestinal permeability also tend to have highly variable plasma exposure profiles within and between individuals [4, 5].

In the design and development of oral drug dosage forms, a PK assessment is fundamental for distinguishing between *f*_abs_ and bioavailability (*F* or BA) [6]. In general pharmacology, *F* represents the fraction of an administered dose of a drug that reaches the systemic circulation in intact form (parent drug) and it is one of the primary PK properties of any drug product. It is assumed that the bioavailability for any drug product given intravenously is 100% and the bioavailability of any orally administered dosage form is measured relative to this value, calculated as the ratio of dose-normalized plasma exposure.

Many of the drug candidates currently generated in early development are lipophilic. This is probably a result of the drug discovery lead-finding techniques applied, which rely on *in silico* and *in vitro* screening of receptor-ligand interactions [7-9]. These molecular screening tools propose candidates with a high potential for interacting with designated target receptors. As these receptors are usually associated with lipophilic drugs, the tools invariably select lipophilic candidates [10-12]. While lipophilic active pharmaceutical ingredients (APIs) tend to have more than sufficiently high effective permeability across the apical membrane of the enterocytes, they suffer from limited solubility and dissolution in the GI lumen [13].

**Figure 1.**
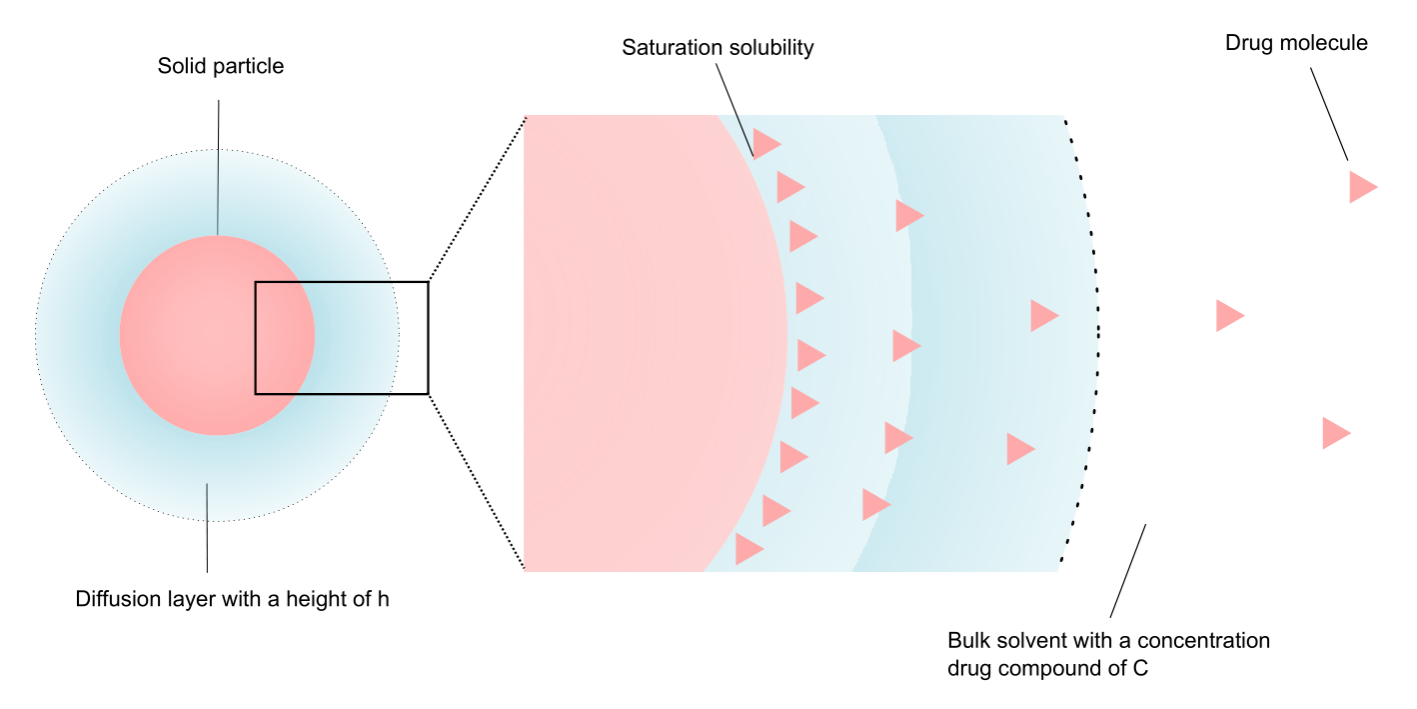
Dissolution of a solid API releases the drug molecule, shown here for an unrestricted volume.

The dissolution process of a solid API particle can briefly be described in two steps. First, the drug molecules are released from the particle surface to the surrounding dissolution media, which creates a saturated, stagnant layer adjacent to the solid surface of the particle. Thereafter, the released drug diffuses into the bulk of the solvent from regions of high, to regions of low, drug concentration (schematically displayed in Figure 1).

The rate of drug dissolution (d*M*/d*t*) is traditionally described by the Noyes-Whitney/Nernst-Brunner equation 1 [14-18]:


(1)

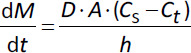



where d*M*/d*t* is the dissolution rate (i.e., the change in dissolved drug concentration with time), *A* is the surface area (4π*r*^2^) of the particle available for dissolution, *D* is the diffusion rate constant, *h* is the thickness of an aqueous boundary layer (ABL) with limited convection surrounding the particle, *C*_s_ is the saturation solubility of the drug at the particle-dissolution media interface, and *C*_t_ the dissolved drug concentration in the bulk at time *t*. The equation assumes that the diffusion of an API monomer through the ABL is the slowest (i.e., rate-limiting) step in this series of events. This assumption allows the incorporation of Fick’s law of diffusion to quantify the dissolution rate of a solid in its own solution (Figure 1) [19].

The Noyes-Whitney/Nernst-Brunner equation (Equation 1) predicts the dissolution of particles greater than a few micrometres; however, this equation may not be optimal for nanoparticles [16]. In addition to increasing total surface area, it should also be mentioned that particle size reduction (down to about 1 μm in diameter) will affect the ABL surrounding each particle, both according to the Prandtl equation (Equation 2) but also by a reduced influence of convection [20, 21].


(2)

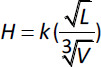



In Equation (2) *H* stands for the hydrodynamic boundary layer thickness, *k* is a constant, *L* is the length of the particle surface in the flow direction, and *V* is the relative velocity of the liquid surrounding the particle. Theoretically, a reduced particle size also increases the saturation solubility, according to the Kelvin effect [20]. The theorem originates from the description of vapour pressure over a droplet, which increases with droplet curvature. This theorem originates from the description of vapour pressure over a droplet, which increases with droplet curvature. The true implication of this effect is however difficult to assess. First, as the effect apply to particles below 10 nm only a fraction (<1%) of the original mass of a conventional nanosized (~100 nm) system remains to be dissolved. Secondly, in polydisperse particles systems, the gained dissolution of small particles may be countered by growth of larger particles, according to the Ostwald ripening principles.

Dissolution is typically the key parameter for the assessment of the onset of action of an oral dosage form with BCS class II drugs. The dissolution rate of any oral drug product is determined by the physicochemical properties of the substance, dosage form composition and functions, as well as by the physiological GI conditions (Table 1). The latter varies within and between subjects as well as between the fasted and fed state.

A range of formulation strategies to increase the dissolution rate have been invented and established for oral drug products with APIs of low solubility [16]. For acidic and basic APIs, a common approach is to form a salt with the charged form of the API. Use of a counter-ion increases the solubility and dissolution rate in the diffusion layer around the solid drug particles [22]. Three other strategies are to: (i) predissolve the drug in a lipid-based formulation for circumventing the slow dissolution step; (ii) change the solid state properties of the APIs from stable crystalline state(s) to metastable amorphous structures with lower intermolecular cohesive forces; or (iii) reduce the particle size, which increases the surface area of the API and consequently the dissolution rate [23-25].

Two established pharmaceutical strategies to reduce the size of the solid state particles are to create micro- or nanosuspensions of them [26]. Particle size reduction (i.e., micronization) enhance the dissolution rate (Equation 1) and may consequently increase the intestinal absorption and bioavailability of low solubility drug compounds. However, for BCS II APIs, nanosuspensions appear to offer additional advantages over microsuspensions; the enhancement of the *in vivo* dissolution rate required to increase the GI drug absorption and bioavailability for low solubility drug candidates is often greater than what a nanosuspension can achieve [27]. This is shown for a nanosuspension of a test drug with low molecular mass 450, acid p*K*a 4.7, log *P* of 5 and a solubility of 2 μM at pH 6.8 [28]. Interestingly, with these nano-technologies for solid-state particles, it is apparent that additional intraluminal mechanism(s) than dissolution must be operating [29].

Despite the advantages with nanoparticles, a number of hurdles need to be resolved with these oral drug delivery systems. For instance, instability related to aggregation of nanoparticles and/or to a change in the solid-state properties of the API may reduce their applicability in pharmaceutical development [30]. Still, in oral product development, nanotechnologies have been successful to develop products to a clinical stage based on solubility, but mainly by enhancing dissolution. However, there is a need to fully understand the contribution from enhancement of solubility and/or dissolution as well as additional absorption mechanisms, such as particle drifting in the mucus layer [31]. There are even strategies in which nanoparticles may increase absorption of low permeability compounds through increased transepithelial transport [32]. Obviously, more formulation research and development beyond the established pharmaceutical strategies are needed, to better understand the mechanisms, physiological possibilities, and limitations in the *in vivo* GI absorption of orally administered nanoparticles.

This review focuses on nanoparticle-based oral drug delivery systems and their *in vivo* performance based on experimental studies in single-pass intestinal perfusion (SPIP) models. In particular, the intestinal absorption mechanisms will be discussed. These data may contribute to a future framework for enabling oral formulation strategies for mainly BCS class II drugs. This review looks at the role of nanoparticles of four low solubility-high permeability drugs: aprepitant, fenofibrate, cyclosporine, and danazol. A summary of data from both fasted and fed simulated conditions is included, because prandial state has a strong effect on rate and extent of intestinal drug absorption for some drug products. Prandial state also illustrates some of the different intraluminal dissolution and absorption mechanisms that come into play.

## Intraluminal transport of nanoparticles and drugs in the intestine

Pharmaceutical development of oral drug delivery systems for low solubility drugs frequently uses particle size reduction as a formulation method. Development of particle sizes in the nano-range (diameter <1000 nm) for oral products has increased as a result of improvements in pharmaceutical processing and manufacturing [33]. As discussed above, oral nanosuspensions dissolve faster than microsuspensions, and accordingly increase intestinal absorption and bioavailability of BCS II compounds [28, 34-37]. Theoretically, API particle size reduction reaches a point at which dissolution is no the longer the rate-limiting step in *in vivo* absorption. At that point, further size reduction is not expected to increase intestinal absorption as it has become solubility controlled [8]. However, experimental observations indicate otherwise. Absorption rates continue to increase with decreasing particle size, which suggests that additional absorption-promoting intestinal processes are taking place simultaneously [29, 38, 39]. It has been proposed that nanoparticles are crossing the epithelial mucus layer to a greater extent than larger particles [40]. Mucus is predominately comprised of the glycoprotein mucin and water. This gives it hydrophilic and hydrophobic domains, a negative net charge, and high porosity and pore interconnectivity. In addition, mucus is a dynamic, semipermeable transport barrier that is continuously secreted, shed, and digested.

Mucus thickness is about 120 μm, 480 μm and 830 μm in the jejunum, ileum and colon, respectively, with a turnover time of a few hours [41, 42]. The small intestinal mucus contains a single mucus layer that is not a rate-limiting step in the absorption, not even for lipophilic high-permeability drugs and/or high permeability drugs transported by an efficient carrier-mediated (CM) transport route [43-45]. Based on *in vivo* jejunal perfusion at different rates in humans, the resistance of the mucus layer to the intestinal absorption of highly permeable solutes is markedly overestimated. Instead, intestinal absorption in humans seems to be membrane controlled for both low- and high-permeability compounds, irrespective of transport mechanism [46]. It is well-recognized that nanoparticles interact with mucus by different mechanisms and that nanoparticles may acquire different biological and physicochemical properties as a consequence of adsorption of intraluminal GI biomolecules to their surface (i.e., corona formation).

Commercially available oral nanoparticles are typically around 100–300 nm, which should allow them to pass faster than larger API particles across the gel-like, mucin-based mesh structure composing the mucus layer. A continuous mucus layer exists throughout the small intestine, and is highly stratified adjacent to the epithelium. Experiments show that mucin pore size is consistent with free diffusion of 100 nm particles, but limited diffusion of 500 nm particles [40, 47]. This is due to increased diffusivity for a spherical particle with smaller radius, which then establishes a high local drug concentration available for permeation adjacent to the apical enterocyte membrane [31, 48]. The primary mechanism of transport through this layer is diffusion, as opposed to the intestinal lumen, where the primary transport mechanism is convection [49]. The combined effects of increased dissolution, mucus penetration, and mucus layer diffusivity for small particles seem to explain the increased intestinal absorption of nanoparticles compared to larger ones.

## Method of *in vivo* absorption: single-pass intestinal perfusion model and absorption calculations

**Figure 2.**
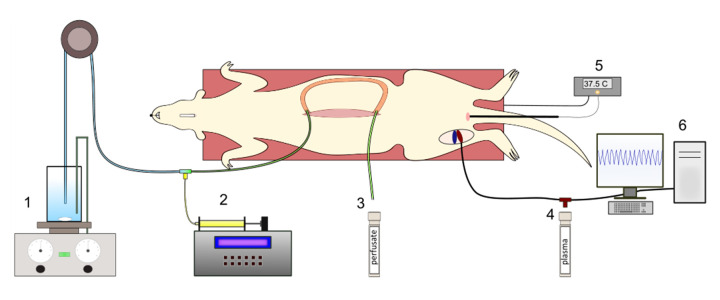
Graphical illustration of a single-pass intestinal perfusion (SPIP) in rat with the intestinal segment placed on the outside of the abdomen. The different numbers show: 1) perfusion solution/suspension under constant stirring on a heating table, and perfused using a peristaltic pump; 2) a syringe pump allows co-administration of e.g., enzymes; 3) perfusate is collected after passing through an intestinal segment; 4) blood sampling from the femoral artery; 5) body temperature is monitored using a rectal probe, connected to a heating pad; 6) blood pressure and heart rate are monitored in the femoral artery.

This review discusses mainly data from the rat and pig single-pass intestinal perfusion (SPIP) models in relation to other relevant *in vivo* data [50-53]. The SPIP model has been extensively used in preclinical studies to investigate and determine epithelial effective intestinal permeability (*P*_eff_), as well as other absorption parameters for drugs and drug delivery systems, such as suspensions and lipid-based ones (Figure 2). Intestinal *P*_eff_, absorption flux (*J_app_*), and fraction dose absorbed (*f*_abs_) are the three key biopharmaceutical variables that describe the absorption and transport properties of a drug across the intestinal barrier in the SPIP model [3, 54]. This model is used to investigate oral drug delivery and characterize pre-formulations, as part of the development process of a pharmaceutical product. The SPIP model is also useful for investigating regional intestinal permeability and transport mechanisms involved during *in vivo*–relevant conditions, and for determining the BCS classification of any API [54]. This model is also used to investigate the interplay between *in vivo* dissolution of various dosage forms, such as nano-formulations and various pharmaceutical excipients, and intestinal permeability, during both fed and fasted conditions [55].

In the SPIP model, absorption parameters can be calculated in several ways. One of the most common is to calculate effective permeability (*P*_eff_), which is based determining the disappearance of an API from the intestinal lumen during single-pass perfusion of a well-defined intestinal segment (Equation 3):


(3)

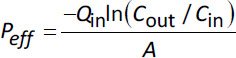



where *Q*_in_ is the perfusate flow rate, *C*_out_ (corrected for water flux) and *C*_in_ are the concentrations of API leaving and entering the intestinal segment, and *A* is the surface area of the perfused intestinal segment, which is assumed to be a smooth cylinder [56]. The *C*_out_ is corrected for water flux using an unabsorbable marker and by weighing the luminal perfusates leaving the segment [57].

The disappearance flux (*J*_disapp_) of an API can also be calculated from perfusate data leaving the intestinal segment by correcting for water flux as previously described (Equation 4):


(4)

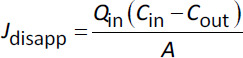



Alternatively, absorption flux (*J*_app_) can be calculated in the SPIP model by monitoring plasma drug appearance using the deconvolution method (Equation 5). However, this requires a separate intravenous injection of the study drug for calculating the disposition PK parameters in the deconvolution.


(5)

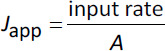



The pig data in this review are based on the same perfusion setup as the Loc-I-Gut model, for which it has been clearly shown that a well-stirred model describes the hydrodynamics during single-pass experiment [56, 58]. The following calculations from the perfusion experiments in the pig SPIP model were made from steady-state concentrations of the outlet jejunal perfusate [55]. The fraction of the drug absorbed in the segment during the perfusion (*f*_abs_) was calculated from Equation 6:


(6)

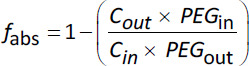



where *C*_in_ and *C*_out_ are the concentrations of the study drug, and PEG_in_ and PEG_out_ are the non-absorbable volume correction marker ([^14^C]-PEG 4000), entering and leaving the jejunal segment, respectively. The jejunal *P*_eff_ of each drug is calculated according to a well-mixed tank model, as shown in Equation 7 [59]:


(7)

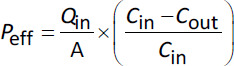



where *A* is the cylindrical area of the perfused jejunal segment (A).

## *In vivo* absorption of four model drugs, under different luminal conditions

This review focuses on the *in vivo,* small intestine absorption of four BCS class II drugs: aprepitant, cyclosporine, danazol, and fenofibrate. A range of physicochemical properties of these drugs are presented in Table 2. They have been investigated in the small intestine as nano-formulations under different luminal conditions.

Aprepitant is an orally administered neurokinin NK-1 receptor antagonist, used clinically to prevent acute and delayed chemotherapy-induced nausea and vomiting [61]. The molecular mass is 534 Da. It has a basic p*K*a of 2.4, an acidic p*K*a of 9.2, and is primarily uncharged at a jejunal pH of 6.5. The aqueous solubility is poor (0.37 μg/mL) and it has a high apparent permeability (*P*_app_) in Caco-2 cells (1.7·10^−4^ cm/s), which predicts a high *P*_eff_ in both small and large intestine [62]. In fact, the rat small intestinal *P*_eff_ is reported to be 1.7·10^−4^ cm/s [63]. Aprepitant is mainly metabolized by gut and hepatic CYP3A4, which means that plasma pharmacokinetics (i.e. F) can be affected by differences in small intestinal absorption rate [64]. The oral product with aprepitant (Emend; Merck & Co., Inc., NJ) is based on nanoparticles of the API and has an average particle diameter below 200 nm. These particles are coated onto larger cellulose beads and encapsulated [65].

Cyclosporine is an immunosuppressant used to prevent organ transplant rejection and danazol is a steroid used to treat endometriosis. As evident by their BCS class II classification, both cyclosporine and danazol have high intestinal permeability values and low aqueous solubility.

Both cyclosporine and danazol are metabolized in the small intestine and liver by CYP3A4, where the gut wall and liver first-pass extraction might be affected by differences in small intestinal absorption [66-68]. The size of the nanoparticles of cyclosporine and danazol used in the experiments discussed below was 650 and 150 nm, respectively. To ensure that the nanosuspension was homogenous and stabilized, cyclosporine and danazol were added to an aqueous solution containing small amounts of water-soluble polymer and surfactant as stabilizers/dispersants (PEG-4000 0.13 mg/mL, monoolein 4 mg/mL, sodium taurocholate 20 mg/mL, oleic acid 20 mg/mL, and phosphatidylcholine 6 mg/mL). Both model drugs were single-passed perfused through pig jejunum in isotonic fluid alone with and without a P-gp inhibitor, and with dietary and endogenous lipids. To determine the effect of food on the *in vivo* dissolution of cyclosporine and danazol, saturated drug solutions in isotonic fluid containing lipids were also perfused in pig.

Fenofibrate is mainly used for primary hypercholesterolemia or mixed dyslipidemia. Fenofibrate is a low molecular mass (360.4 Da) BCS class II drug that is very lipophilic (log *P* = 5.24), with an aqueous solubility of <0.1 mg/mL. Fenofibrate is an ester of fenofibric acid. After oral administration in humans, it is completely converted to its active metabolite, fenofibric acid. Fenofibric acid and fenofibric acid glucuronide are then excreted into urine (60% of the dose) and feces (25%) [69].

## Comparison between micro- and nanosuspensions of BCS class II drugs

Experimental data and simulations show that small intestinal absorption of larger particles of danazol (226 μm), griseofulvin (118 μm), and aprepitant (26 μm), is dissolution rate-controlled [70]. Takano *et al*. also show that reduction of particle size increases *in vitro* dissolution and improves intestinal absorption in dogs. Increasing the dissolution rate by using of smaller particles of these three selected model drugs does not improve their small intestinal absorption because their absorption is limited by their solubility during non-sink luminal conditions [70]. However, for some drug formulated as nanoparticles in the range of 50–200 nm, the absorption and plasma exposure increased. The increased absorption rate is often explained by enhanced drug dissolution rate [28, 34, 71]. These differences in experimental *in vivo* data and their interpretation will be discussed below.

The intestinal absorption rate of aprepitant increases by increasing the concentration of nanoparticles (200 μM versus 20 μM) in the perfusion suspension passed along the small intestine segment in the rat SPIP model [5]. Aprepitant permeates the apical membrane as a monomer very swiftly and has a small intestinal *P*_eff_ of 1.7 · 10^-4^ cm/s. Both the drifting and deposition of nanoparticles with aprepitant in the ABL, as well as colloidal structures, may contribute to an increase in intestinal absorption. Absorption of nano- and microsuspensions has been compared in the rat SPIP model when the small intestinal segment was perfused with buffer, FaSSIF, and FeSSIF. In with single-pass perfusions of jejunum, the plasma concentration time–curves clearly show that fed conditions, i.e. FeSSIF, increase the intestinal absorption of aprepitant as microsuspensions, but not as nanosuspensions. This difference is in line with previous data from both dog and human, in which nanosuspensions seem to prevent variation in plasma exposure between different prandial states, while microsuspensions show significant food-effects [72-75].

The mucus layer also influences absorption of drugs delivered orally as nanoparticles [76]. For example, the hydrophobicity, electrostatic properties, and steric hindrance, of mucus are key feature that prevents the hydrophilic pancreatic proteases from acting on, and thereby injuring, the intestinal epithelium [77]. To quantify the jejunal flux of aprepitant across the enterocytes, *J*_app_ was calculated according to Eq. 4 (Table 3). There were no significant differences for micro- and nanosuspensions, but a large, albeit non-significant one, for the microsuspensions in FeSSIF compared to buffer and FASSIF (64 and 4.8 times higher, respectively). For the nanosuspensions, the *J*_app_ were all within a 1.6-fold range, reiterating the effective elimination of a luminal food effect by the nanoformulations. The high variability of the data in this paper demonstrates that studies with more animals are needed to verify these observed trends for nano- and microsuspensions (Table 3).

## *In vivo* absorption of BCS class II drugs in nanoparticles at different luminal conditions

### Danazol and cyclosporine

Intestinal absorption mechanisms have been investigated in fed and fasted states in a pig SPIP model for low solubility APIs as monomers or nanoparticles. Nanoparticle suspensions of two BCS class II model drugs, danazol and cyclosporine (CyA), were single-pass perfused through the pig jejunum in isotonic buffer alone (control) and isotonic buffer with verapamil, a P-glycoprotein (P-gp) inhibitor, or in isotonic buffer containing dietary and intestinal lipids. Co-administration of CyA and verapamil increased the jejunal *P*_eff_ of CyA from 0.63 ± 0.05 ∙ 10^-4^ cm/s to 1.01 ± 0.09 ∙ 10^-4^ cm/s [55]. The human jejunal *P*_eff_ for CyA was  1.65 ± 0.53 ∙ 10^-4^ cm/s, when determined with the perfusion method, which classifies CyA as a low solubility-high permeability Class II BCS drug. CyA is well-recognized to have highly variable absorption from approved oral formulations because of poor dissolution [54, 79, 80]. When dietary lipids were introduced to the perfused segment in the SPIP model, they reduced the jejunal absorption of a CyA as a saturated solution and nanosuspension by approximately 50% and 83%, respectively (Table 4). In contrast, dietary lipids increased the jejunal absorption of danazol nanosuspensions more than two-fold.-However, *f*_abs_ was reduced by 60% when danazol was administered as a solution in the same media (Table 4).

This difference for nanosuspensions and monomer solutions of danazol is most likely due to increased dissolution and/or a more efficient transport of the danazol nanoparticles (150 nm) than the CyA (650 nm) through the particle ABL. The difference in the effect of intraluminal lipids may also be explained by different distribution of any of the study drugs to the colloidal structures present in the media. These structures affect the convective and diffusion rates of the drugs differently in the perfused segment. CyA has a higher partitioning (2.5 times) to the colloidal structures than danazol. Based on previous observations, danazol probably partitions primarily to the mixed intraluminal micelles, and to a lesser extent to the lipid vesicles [55].

Despite a lower log *P* value, CyA most likely partitions into larger lipid vesicles to a greater extent than danazol. It has a larger molecular size, which results in a reduced thermodynamic activity (i.e., lower free concentration available for permeation). Furthermore, there would be an overall slower diffusion and convection in the intestinal lumen. Another plausible mechanism is that the slow and incomplete partitioning from the vesicles to the aqueous-based chime leads to a subsequent rapid intestinal absorption of CyA. A temporarily unsaturated aqueous phase might form along the perfused jejunal segment and reduce thermodynamic activity. It is also clear that drug-solubilization is a more important factor in fed state for CyA than P-gp inhibition for food–drug interaction. Drug partitioning into different luminal CS, and their fate in the lumen and across the ABL, are important processes to consider in designing nano-based oral formulations of poorly soluble drugs.

Both the *P*_eff_ and *f*_abs_ were higher for danazol when administered as a nanosuspension in lipid-containing media than in the control buffer. However, due to the rapid dissolution of the nanoparticles, the difference in absorption did not correspond to previously observed increases in bioavailability of danazol administered with food. Administration of danazol in the same media, but as a solution, decreased both *P*_eff_ and *f*_abs_. This shows the importance of the rapid dissolution of the drug nanoparticles. When danazol is administered as a solution, thermodynamic activity may decrease because absorption of the drug might create a state of undersaturation.

### Aprepitant

The solubility of aprepitant in biorelevant media, e.g., fasted-state simulated intestinal fluid (FaSSIF), is approximately 60 times higher than in buffer alone [60]. This indicates that aprepitant readily partitions to luminal colloidal structures. The influence of these colloidal structures on the *in vivo* absorption of aprepitant was investigated in the rat SPIP model. When colloidal structures were added in the perfusion medium, the absorption was indeed affected as the observed small intestinal absorption rate increased. However, the experimentally determined increase in small intestinal absorption rate cannot be explained only by an increased luminal dissolution rate. Rather, the increased absorption rate may be a consequence of changes to the total effective diffusion from the bulk to the epithelial membrane of aprepitant, i.e., transport as free monomers and monomers partitioned to colloidal structures, and as particles [63]. Total effective diffusion may be an appropriate parameter to describe these experimental observations and to translate the SPIP data on nanoparticle formulations to *in vivo* performance. These specific data have been used to evaluate a mathematical model simulating the absorption-promoting effects of nanoparticle formulations. This model included interlinked descriptions of hydrodynamics, particle dissolution, and particle diffusion for both the API and luminal colloidal structures. This mathematical, mechanistic model adequately describes the above discussed *in vivo* absorption data of nano-formulated aprepitant. It also supports the proposed mechanism by which contributing effects of the diffusion across the mucus layer of both aprepitant nanoparticles and colloidal structures into which the drug had partitioned [81]. This exemplifies a model in which representation of physiology and description of mechanistic formulation-physiology relationships was pivotal to establish the role of formulation for effective drug absorption. The need of such physiologically based biopharmaceutics models (PBBM) for predictions and assessments of formulations *in vivo* performance has recently been highlighted with an increased scientific activity in the area [1, 82].

### Fenofibrate and other drugs

In contrast to these absorption-promoting results for nanoparticles aprepitant and danazol in both fasted and fed conditions in the SPIP model, only the fed state reduces the intestinal absorption of nano-sized drugs such as fenofibrate, CsA, pafenolol [37, 55, 83, 84]. In a regional absorption study in humans with a site-specific delivery system (Enterion capsule), fenofibrate were given as single, equimolar doses into the stomach, proximal small bowel, distal small bowel, and colon. The bioavailability (related to an intravenous dose of fenofibric acid) in the stomach, proximal small bowel, distal small bowel, and colon was 69%, 73%, 66%, and 22%, respectively [85]. This clearly shows that fenofibrate is absorbed from the small intestine. However, the solubility and dissolution conditions in the colon—less fluid and limited amounts of bile acids and colloidal structures—prevent efficient fenofibrate absorption from this region.

An *in vivo* study in rats compared in vitro dissolution, solubility and bioavailability of three different oral nanosystems with fenofibrate powder. The three systems included PVP nanospheres, HP-β-CD nanocapsules, and gelatin nanocapsules [86]. the most improved apparent solubility and oral bioavailability in fasted rats was with fenofibrate nanoencapsualted with gelatin at a ratio of 1:8 (w/w). The authors of the study proposed that the improved dissolution rate and subsequent intestinal absorption was due to: (i) a higher solubility of fenofibrate as it converted into the amorphous form or nanocrystalline state; (ii) a large surface area for dissolution of drug; (iii) improved wetting of fenofibrate by the polymeric matrix in the formulation; and (iv) reduction in the crystalline intensity.

A method to investigate *in vivo-*relevant mechanisms of drug absorption in humans is by GI fluid sampling [52, 87]. GI intubation can explore directly the dynamic interplay of drug release, dissolution, precipitation, and absorption of the drug from a dosage form. The dissolved drug and solid-state concentration–time profiles from different segments in the GI tract can be obtained. In addition, direct infusion of a suspension into the duodenum allows patient control of the therapeutic system [88, 89].

One study compared nano- and microsuspensions of fenofibrate administered orally in fed state in humans. The nanosuspensions gave higher local concentrations than the microsuspensions in the proximal small intestinal lumen and resulted in higher absorption and plasma exposure–time profile of the drug [37]. In fed conditions, the duodenal concentrations of fenofibrate were higher for both oral formulations, but there was no increase in plasma exposure of fenofibric acid. Micellar encapsulation of the fenofibrate in the lumen may have limited potential to permeate from colloidal structures in fed-state intestinal fluids. Absorption can also decrease in the presence of micelle-forming lipids due to lower thermodynamic activity [39]. The absorption of BCS class II drugs does not necessarily increase when administered with food, as proposed by others [90]. Taken together, these *in vivo* data demonstrate that increasing the intestinal luminal concentration of BCS class II drugs in complex intestinal fluids does not always result in more rapid and extensive intestinal absorption. Especially in fed state, the encapsulation of a lipophilic drug in micelles and vesicles in the intestinal lumen may reduce the potential for intestinal flux.

## Conclusion

The use of oral, nanosystems for drug delivery can increase intestinal absorption and bioavailability, reduce the risk for food–drug interactions, and pH-dependent intestinal absorption. However, for some drugs, the interaction with colloidal structures in the lumen may prevent absorption even if the oral nanosystems enhance dissolution *in vitro* compared to the standard oral formulation. Nevertheless, nanoparticle formulations offer an important strategy in the development of new drugs and for currently difficult poorly soluble drugs.

## Figures and Tables

**Table 1.** Overview of the pharmaceutical, physicochemical and the physiological properties that influence intestinal drug dissolution.

Properties	Affected by		
	Physicochemical parameters	Pharmaceutical parameters	Physiological parameters
Drug particle surface area		Wettability, particle size, aggregation, surfactnts	Deaggregation by surfactants in gastric juice and intestinal fluid (from bile)
Drug diffusion	Molecular size (Stokes–Einstein equation)		Viscosity of GI luminal contents (fasted/fed) (Stokes–Einstein equation)
Diffusion layer	Surface energy	Particle size	Motility patterns and luminal flow rate
Drug solubility	Lipophilicity, pKa, melting point	Crystal form, solubilisation, precipitation, pharmaceutical excipients, impurities	Luminal pH, buffer capacity, bile and food composition
Amount of drug dissolved	See above	See above	Intestinal permeability
Volume of solvent available			GI secretion, fluid absorption, co-administered fluids

**Table 2.** Physicochemical descriptors of danazol, cyclosporine, aprepitant, and fenofibrate.

	**danazol** [55, 60]	**cyclosporine** [55]	**aprepitant** [60]	**fenofibrate** [60]
Molecular mass (g/mol)	337	1202	535	361
Water solubility at 37 °C (μg/ml)	0.5	7	0.37	0.25
log *P*	3.7	3	4.7	6.9
*P*_app_ (·10^-6^ cm/s)	14.15	2.6	170	220

**Table 3.** Historical *in vivo* absorption flux (*J*_app_) (SD) for aprepitant nano- and microsuspensions determined in the jejunum of the single-pass perfused rat reported by Roos *et al*. 2018 [78].

Media	Flux (× 10^-3^ μmol / (hr × cm^2^))	
	Nanosuspension	Microsuspension
Buffer	2.91 (1.02)	0.104 (0.081)
FaSSIF	3.57 (1.97)	1.58 (2.10)
FeSSIF	2.25 (1.69)	6.63 (7.32)

**Table 4.** Mean (± S.D) of effective jejunal permeability (*P*_eff_) and fraction absorbed (*f*_abs_) for cyclosporine and danazol during a single-pass perfusion of nanoparticles, nanoparticles in the fed state, and as a saturated solution.

Parameter	Nanoparticles	Nanoparticles – fed state	Saturated solution
Cyclosporine			
*P*_eff_ (10^-4^ cm/s)	0.63 ± 0.05	0.11 ± 0.14	0.34 ± 0.08
*f*_abs_ (%)	29 ± 2	5 ± 6	13 ± 8
Danazol			
*P*_eff_ (10^-4^ cm/s)	0.9 ± 0.4	2.3 ± 0.4	0.33 ± 0.09
*f*_abs_ (%)	36 ± 10	58 ± 4	17 ± 4
